# Supplementary material for: Powerful gene set analysis in GWAS with the Generalized Berk-Jones statistic
Source: PLoS Genet. 2019 Mar 15;15(3):e1007530. doi: 10.1371/journal.pgen.1007530 (PMC6436759; doi:10.1371/journal.pgen.1007530)
Supplement: S1 Appendix — Explanation of an additional simulation designed to assess the accuracy of the correlation matrix approximation for precalculated summary statistics. (PDF) [file pgen.1007530.s001.pdf]

S1 Appendix. Supplementary Methods

We conduct an additional set of simulations to demonstrate the reliability of the proposed approximation for summary statistic correlation structures when individual-level data are not available. First we investigate the difference between the correlation matrix estimated using individual-level data and the correlation matrix approximated using reference data as described in "Estimation of $\mathbf{\Sigma}$ with precalculated summary statistics." We then compare the difference in p-values when using the two different correlation matrices.

In this set of simulations, genotyping data from the Cancer Genetic Markers of Susceptibility (CGEMS) breast cancer GWAS is treated as the "original" individual-level data. This dataset contains 2,287 women of European ancestry who are genotyped at around 550,000 SNPs with the Illumina HumanHap500 array. From the genotype data we randomly create 2,000 different gene sets, each with 10 randomly chosen genes. For each gene set, we then simulate an outcome according to the model

$$Y_i = \alpha_1 X_{1i} + \alpha_2 X_{2i} + \alpha_3 X_{3i} + \alpha_4 X_{4i} + \alpha_5 X_{5i} + \beta_1 G_{1i} + ...\beta_s G_{si} + \epsilon_i$$

where $i = 1, 2, ..., 2290$, s is a random integer between 0 and the square root of the set size, X_{1i} is distributed as a standard normal random variable, X_{2i} is distributed as a binary variable with mean parameter 0.5, X_{3i} through X_{5i} are the first three principal components calculated from the HumanHap500 data, and the error term is distributed as a standard normal random variable. The effect sizes for the SNPs are $\beta_1 = ... = \beta_s = 0.15$ and all other effect sizes are 1. Standard score statistics are calculated for each SNP in the set using equation (1), and we estimate the correlation matrix using equation (2). The GBJ statistic and p-value are then calculated for the set. This setup mimics the workflow of a researcher performing a standard GWAS with individual-level data and creating summary statistics with two non-genetic covariates (X_{1i} and X_{2i}) and three principal components.

We next act as if we do not possess the individual-level data and are only given the summary statistics from the previous step. Using the same approximation procedure described in the main manuscript, we create three principal components from the European population (CEU) of the 1000 Genomes panel and use those PCs along with the CEU genotype data to approximate the correlation structure in the CGEMS dataset. We then calculate the GBJ statistic and p-value using the summary statistics and approximated correlation matrix. This process mimics the workflow that we use for the main analysis of this paper.

We report several different metrics to evaluate the difference between the approximated correlation matrix and the one calculated using individual-level data. Let $\mathbf{\Sigma}_{CGEMS}$ denote the correlation matrix calculated using CGEMS individual-level data and equation (2). Let $\mathbf{\Sigma}_{1000G}$ denote the correlation matrix calculated using the approximation procedure. Let $\mathbf{A} = \mathbf{\Sigma}_{CGEMS} - \mathbf{\Sigma}_{1000G}$ denote the difference of the two matrices, and let a_{jk} denote the element in the jth row and kth column of $\mathbf{A}$, which is a $d \times d$ matrix. For each of the 2,000 A matrices we calculate the matrix L_1 norm, which is $||A||_{L_1} = \max_{1 \leq k \leq d} \sum_{j=1}^{d} |a_{jk}|$, and we divide by d for scale. We also calculate the Frobenius norm, which is $||A||_F = \sqrt{\sum_{j,k} a_{jk}^2}$, and we divide by d for scale. Finally we calculate the mean and median values of a_{jk} for each A.

S2 Table provides the mean of each of these metrics for all 2,000 simulations. S4 Figure plots the difference in p-values when using the two different correlation matrices. We see from S2 Table that the approximation performs very well in general, as the elements of $\mathbf{\Sigma}_{1000G}$ do not vary much from those of $\mathbf{\Sigma}_{CGEMS}$. The approximated correlation matrix is very similar to the matrix estimated using individual-level data at all different levels of significance. The p-values are also very similar regardless of which correlation matrix is used, and this similarity holds across different set sizes and significance levels. The only exceptions appear to be a handful of p-values calculated at $p \approx 1 \cdot 10^{-10}$ using $\mathbf{\Sigma}_{CGEMS}$ that are given

as $p \approx 1 \cdot 10^{-12}$ when using $\mathbf{\Sigma}_{1000G}$. As explained in the main text, computational limits sometimes introduce inaccuracies for p-values at this extreme level of significance, which is why we generally truncate p-values less than $p < 1 \cdot 10^{-12}$. However these levels lie far beyond the typical threshold used for multiple testing correction, minimizing the practical effect of the difference, which is only observed for a very small percentage of tests. We caution again against reporting p-values less than $1 \cdot 10^{-12}$.
