# Supplementary figures and images for: Powerful gene set analysis in GWAS with the Generalized Berk-Jones statistic
Source: PLoS Genet. 2019 Mar 15;15(3):e1007530. doi: 10.1371/journal.pgen.1007530 (PMC6436759; doi:10.1371/journal.pgen.1007530)

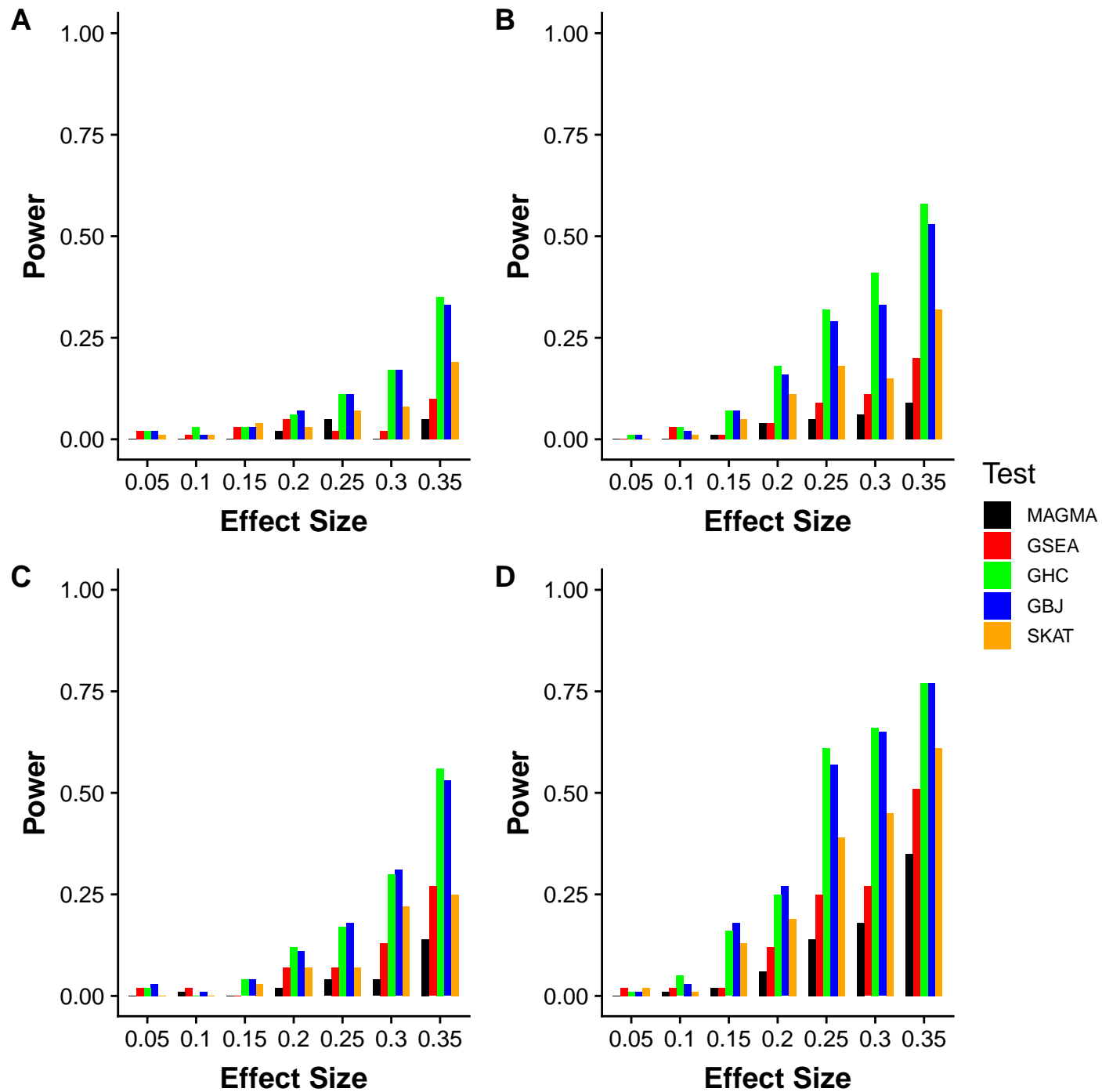

Supplement: S1 Fig — Simulated power of MAGMA, GSEA, SKAT, GHC, and GBJ (all self-contained versions) with random sets of ten genes selected from 10,000 total genes. From the ten genes in the set, a genes are selected to hold b causal SNPs each. The four subfigures correspond to (A) a = 1, b = 1, (B) a = 1, b = 2, (C) a = 2, b = 1, (D) a = 2, b = 2. The effect size is given on the x-axis. We perform 100 simulations at each parameter setting and test at α = 0.01. GBJ and GHC perform well across these very sparse settings. (PDF) [file pgen.1007530.s002.pdf]

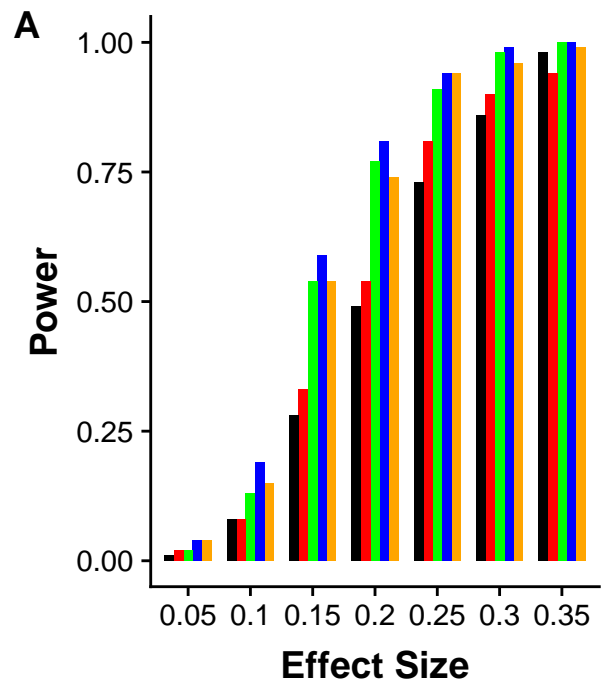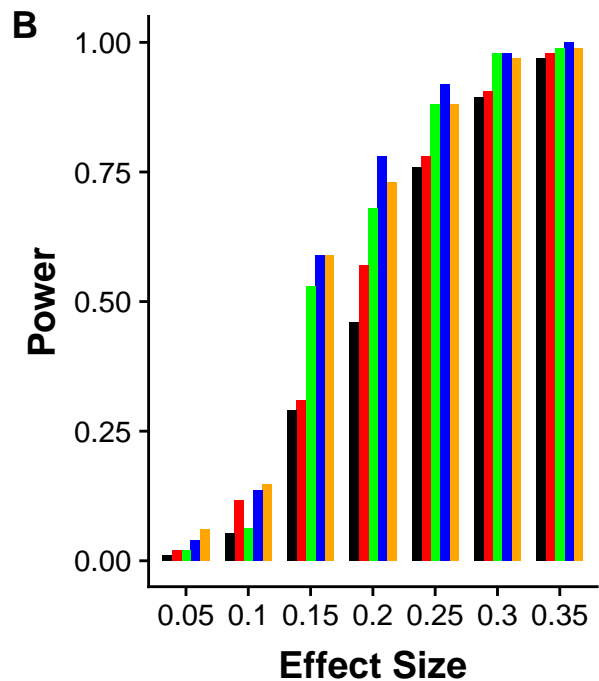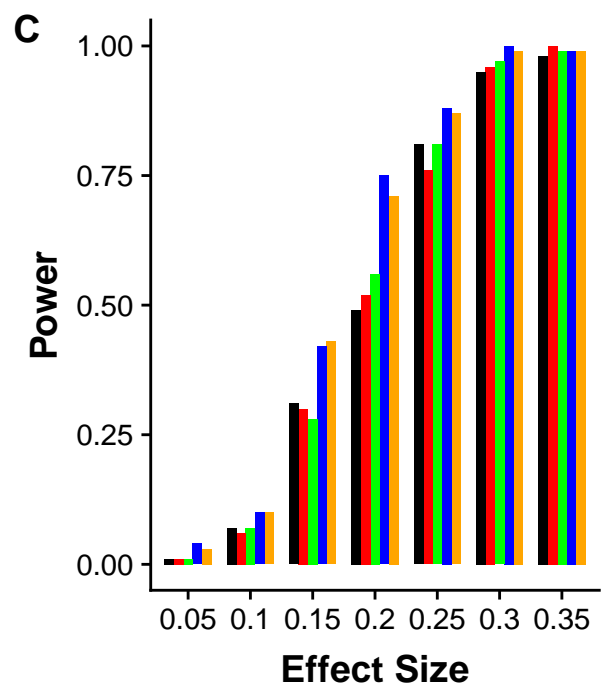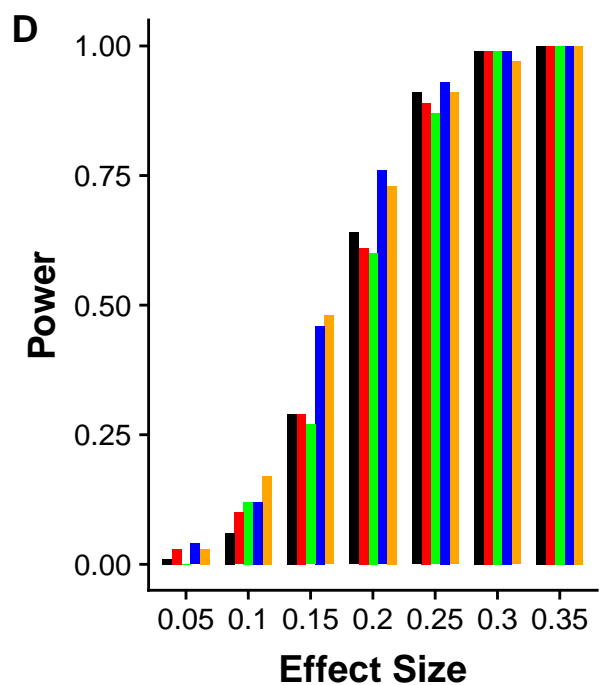

Test

- MAGMA
- GSEA
- GHC
- GBJ
- SKAT

Supplement: S2 Fig — Simulated power of MAGMA, GSEA, SKAT, GHC, and GBJ (all self-contained versions) with random sets of ten genes selected from 10,000 total genes. From the ten genes in the set, a genes are selected to hold b causal SNPs each. The four subfigures correspond to (A) a = 3, b = 4, (B) a = 4, b = 3, (C) a = 6, b = 2, (D) a = 7, b = 2. The effect size is given on the x-axis. We perform 100 simulations at each parameter setting and test at α = 0.01. GBJ and SKAT perform well across these more dense settings. (PDF) [file pgen.1007530.s003.pdf]

Type I Error at  $\alpha=0.01$

0.015  
0.010  
0.005  
0.000

MAGMA

GSEA

GHC

GBJ

SKAT

Test

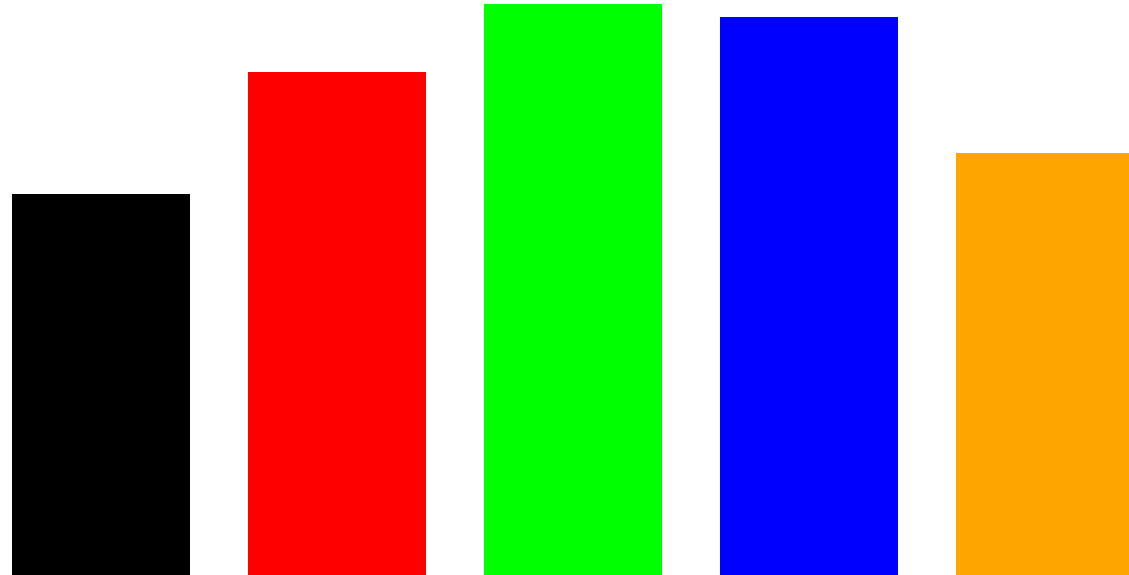

Supplement: S3 Fig — Simulated Type I error of MAGMA, GSEA, SKAT, GHC, and GBJ (all self-contained versions) with random sets of ten genes selected from 10,000 total genes. We perform 4,000 simulations for each method and test at α = 0.01. All tests appear to protect the Type I error adequately. (PDF) [file pgen.1007530.s004.pdf]

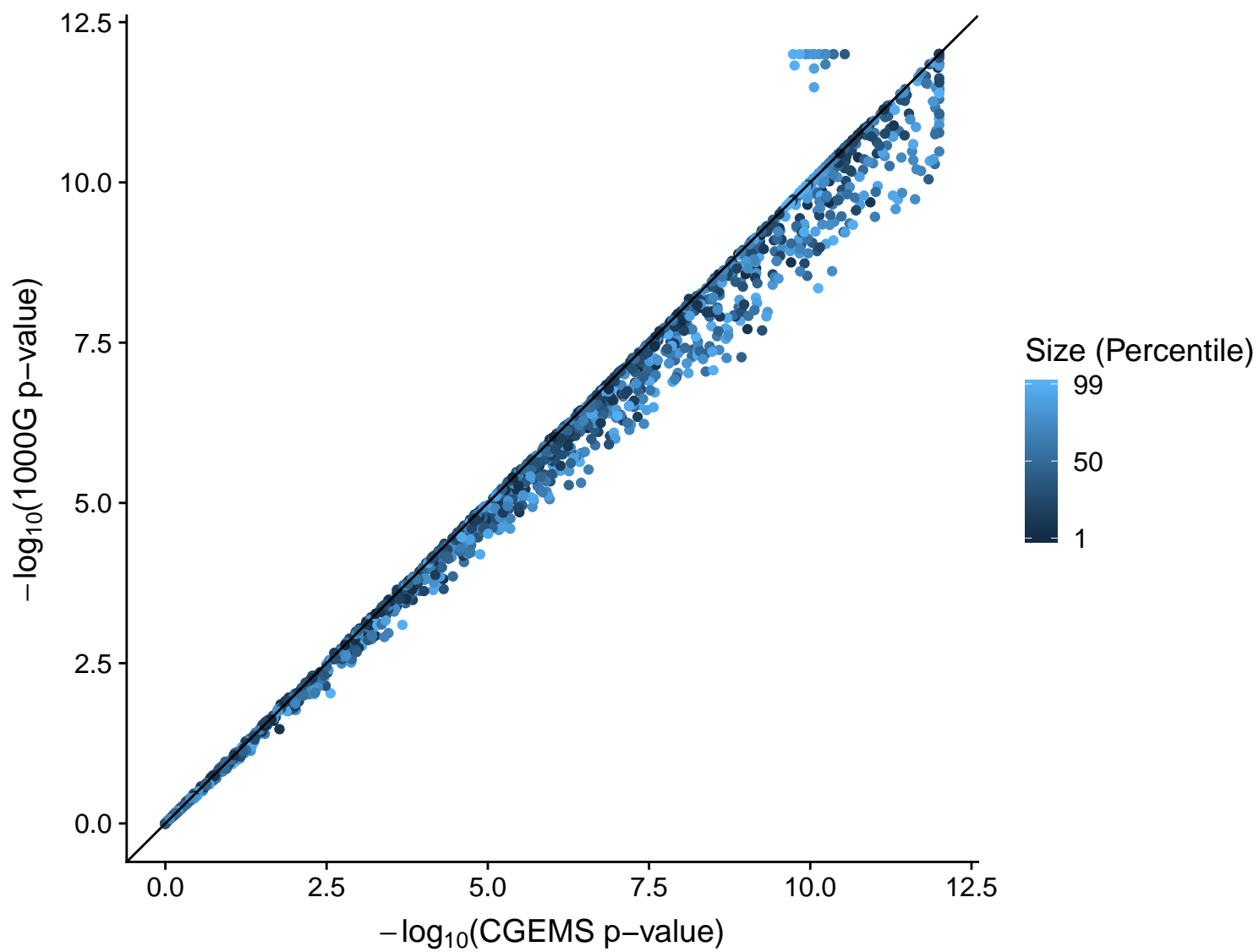

Supplement: S4 Fig — Simulation parameters are given in S1 Appendix. Test statistics are calculated using individual-level data from the CGEMS dataset and their correlation is estimated using the individual-level data and Eq (2). We then estimate the correlation structure again using 1000 Genomes data as detailed in the Methods section. P-values are calculated once with each correlation matrix and compared. In general, the approximated correlation matrix p-values are very close to the p-values calculated with correlation matrices estimated from the original data. When the p-values do differ, p-values calculated with the approximation tend to be slightly more conservative, thus we would expect the approximation to safely protect the Type I error rate. (PDF) [file pgen.1007530.s005.pdf]

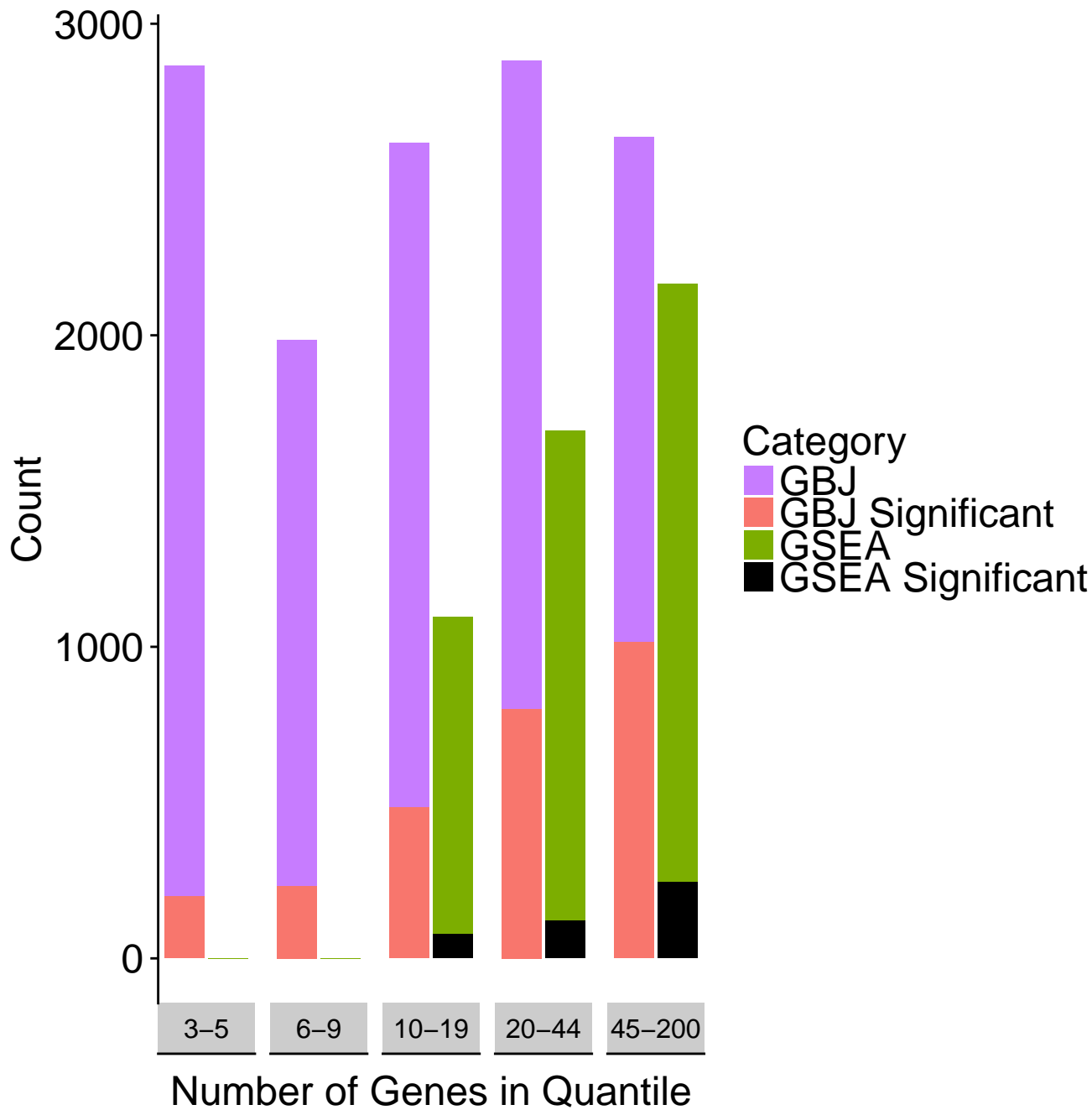

Supplement: S5 Fig — GBJ significance is assigned based on p < 4.65 ⋅ 10−6. GSEA significance is assigned based on permutation-estimated control of the false discovery rate at q < 0.05. GSEA was applied only to pathways with ten or more genes in an effort to reduce false positives and to search for more biologically meaningful results. This choice may omit certain specific and informative functional sets, as pathways with fewer than ten genes account for a large percentage of the pathway database. We investigate these smaller pathways as well with GBJ. The percentage of significant pathways appears to rise as the gene set size increases. This trend is expected, because larger gene sets have a higher chance of including genes such as FGFR2 that will almost automatically drive any pathway to significance. GBJ finds a higher proportion of pathways significant at each of the three gene set sizes tested by both methods. (PDF) [file pgen.1007530.s006.pdf]

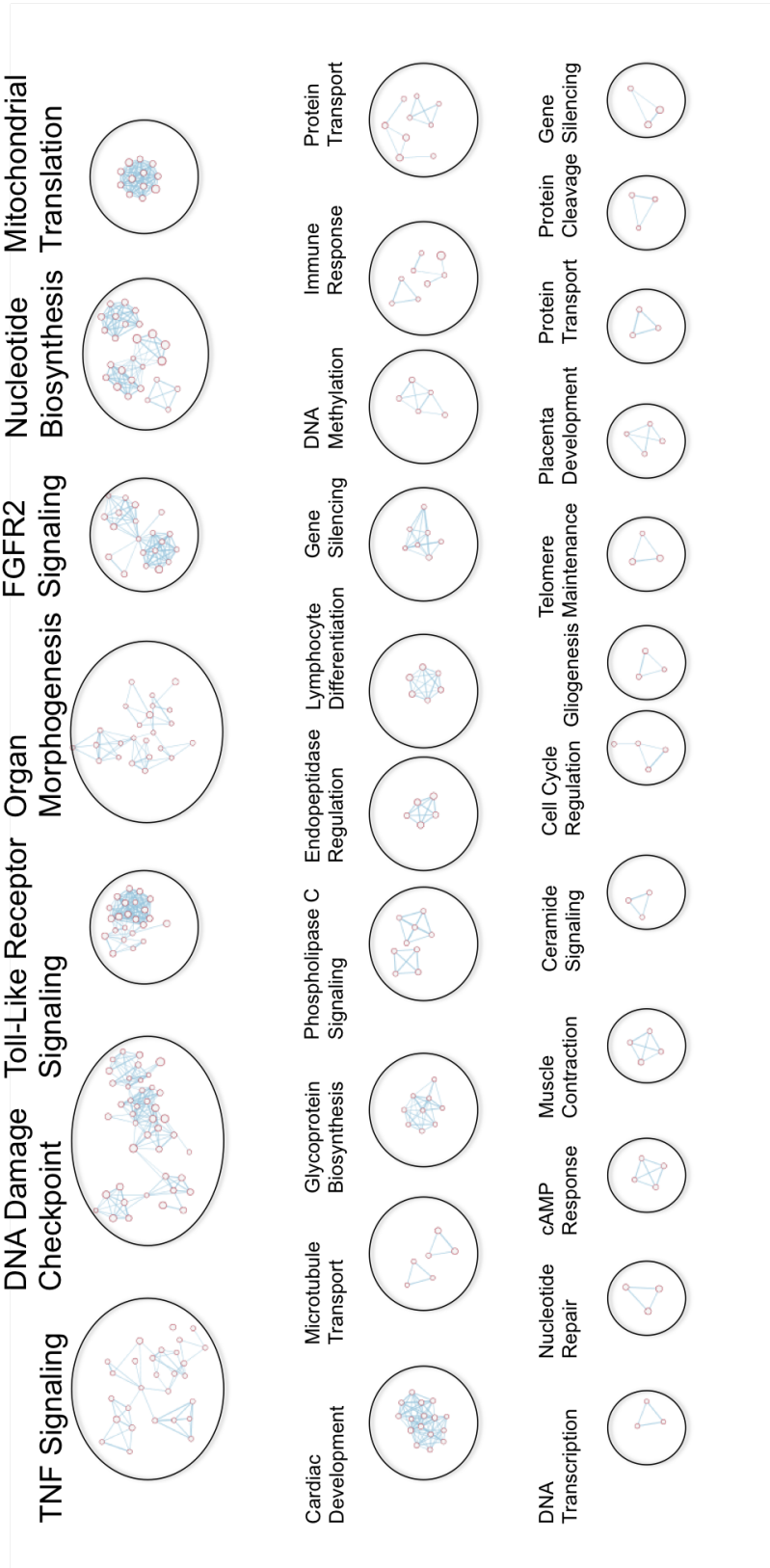

Supplement: S7 Fig — Network created using the EnrichmentMap application in Cytoscape. Each node (red circle) denotes a pathway and each edge (blue line) connects pathways that show overlapping genes. An edge cutoff of 0.5 is used to measure overlap. Only clusters with three or more nodes are shown. Pathways are clustered and annotated by theme. Certain themes such as Organ Morphogenesis and Cell Cycle Regulation were also found in the original GSEA pathway analysis of this data. (PDF) [file pgen.1007530.s008.pdf]
