# Supplementary material for: Powerful gene set analysis in GWAS with the Generalized Berk-Jones statistic
Source: PLoS Genet. 2019 Mar 15;15(3):e1007530. doi: 10.1371/journal.pgen.1007530 (PMC6436759; doi:10.1371/journal.pgen.1007530)
Supplement: S6 Fig — The significance ranking of pathways according to GBJ and GSEA, colored according to density of SNPs with p < 10−5. Pathways are ordered according to p-value or q-value, smaller rankings indicate more significance. Data is identical to that of Fig 4, except here all pathways are shown instead of only those in selected percentiles. Similar to Fig 4, pathways ranked by GBJ as more significant generally have a higher proportion of SNPs with p < 10−5, as there is a clear color trend from blue to red when moving up along the y-axis. In contrast, there is no discernible relationship between a pathway’s GSEA ranking and its density of suggestive signals. (PDF) [file pgen.1007530.s007.pdf]

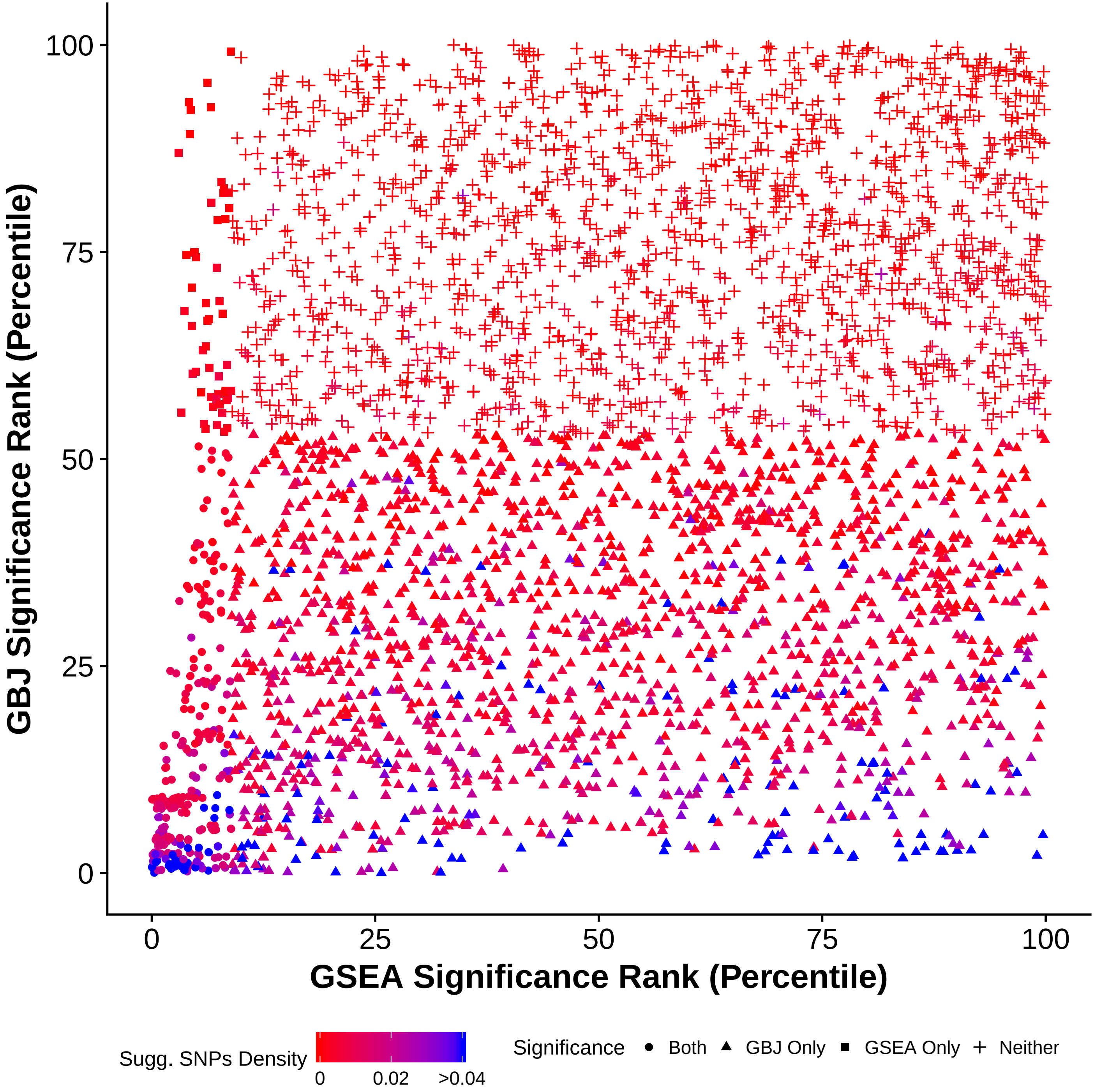
GBJ Significance Rank (Percentile)
100
75
50
25
0
GSEA Significance Rank (Percentile)
0
25
50
75
100
Sugg. SNPs Density
0
0.02
>0.04
Significance
Both
GBJ Only
GSEA Only
Neither
