# Supplementary material for: Powerful gene set analysis in GWAS with the Generalized Berk-Jones statistic
Source: PLoS Genet. 2019 Mar 15;15(3):e1007530. doi: 10.1371/journal.pgen.1007530 (PMC6436759; doi:10.1371/journal.pgen.1007530)
Supplement: S1 Table — Time needed to calculate GBJ and GHC test statistics and p-values. We generate data using the settings of Fig 2C and record the time needed to run GBJ or GHC on one gene set. We stratify the results by high p-value sets (sets demonstrating p > 0.05 using both GBJ and GHC) and low p-value sets (sets demonstrating p < 0.005 using both GBJ and GHC). These times are recorded in the first two columns. We then repeat the timing process except we trim each set to 100 SNPs before testing. These results are recorded in the second two columns. Each column was constructed after timing 2,000 gene sets. GBJ runs faster than GHC in over 99.9% of the simulations. The ratio of running time for GBJ divided by running time for GHC becomes closer to 1 for insignificant sets with high p-values, partly because the analytical p-value calculation requires fewer steps for p-values close to 1. Actual running times may vary depending on computing hardware; displayed times were obtained on a computing cluster where each iteration of the simulation used a single core with 4 GB of RAM. (PDF) [file pgen.1007530.s009.pdf]

| | $d$ random, high $p$ | $d$ random, low $p$ | $d = 100$, high $p$ | $d = 100$, low $p$ |
|---|---|---|---|---|
| Mean GBJ time (sec) | 16.97 | 17.68 | 7.53 | 7.61 |
| Mean GHC time (sec) | 29.60 | 70.13 | 13.06 | 30.88 |
| Mean Ratio | 0.58 | 0.26 | 0.58 | 0.26 |
| Median GBJ time (sec) | 16.90 | 16.72 | 8.41 | 7.54 |
| Median GHC time (sec) | 27.73 | 65.62 | 12.82 | 29.67 |
| Median Ratio | 0.64 | 0.26 | 0.65 | 0.26 |
| Proportion GBJ faster | 0.999 | 1.00 | 0.999 | 1.00 |
