# Supplementary material for: Powerful gene set analysis in GWAS with the Generalized Berk-Jones statistic
Source: PLoS Genet. 2019 Mar 15;15(3):e1007530. doi: 10.1371/journal.pgen.1007530 (PMC6436759; doi:10.1371/journal.pgen.1007530)
Supplement: S2 Table — Simulation results assessing the difference between correlation matrices calculated with individual-level data and those approximated using reference data as detailed in S1 Appendix. We evaluate the difference between the two matrices through the scaled matrix L1 norm, the scaled Frobenius norm, the mean value of all elements in the difference matrix, and the median value of all elements in the difference matrix. We provide the mean of each of these four metrics across all gene sets falling into the designated p-value partitions (according to p-value when using individual-level data correlation matrix). (PDF) [file pgen.1007530.s010.pdf]

P-value Upper	P-value Lower	Matrix L_1 Norm	Frobenius Norm	Mean a_{jk}	Median a_{jk}
1.0000000	0.1000000	0.1111144	0.0761698	-0.0004734	-0.0004113
0.1000000	0.0100000	0.1134069	0.0759688	0.0003394	-0.0003328
0.0100000	0.0010000	0.1019425	0.0698224	0.0000794	-0.0001807
0.0010000	0.0001000	0.1262335	0.0768145	0.0002562	-0.0002737
0.0001000	0.0000100	0.1206222	0.0771068	0.0000303	-0.0003236
0.0000100	0.0000010	0.1254624	0.0790673	0.0005188	-0.0000380
0.0000010	0.0000001	0.1084867	0.0728382	-0.0000042	-0.0003738
0.0000001	0.0000000	0.1197800	0.0756449	0.0001898	-0.0004541
