# Supplementary material for: Powerful gene set analysis in GWAS with the Generalized Berk-Jones statistic
Source: PLoS Genet. 2019 Mar 15;15(3):e1007530. doi: 10.1371/journal.pgen.1007530 (PMC6436759; doi:10.1371/journal.pgen.1007530)
Supplement: S3 Table — Number Tested refers to pathways that eventually contained less than 1,500 SNPs after pruning. Number Significant refers to pathways that demonstrate a p-value less than the Bonferroni-corrected level of p < 4.65 ⋅ 10−6. Height shows many more significant pathways than breast cancer and schizophrenia, supporting previous research demonstrating that height is a highly polygenic phenotype. Breast cancer shows the fewest significant pathways out of all three phenotypes. (PDF) [file pgen.1007530.s011.pdf]

	Breast Cancer	Height	Schizophrenia
Number Tested	10250	10737	10313
Number Significant	2740	7517	3246
Number $p < 10^{-12}$	1106	4916	1447
