# Supplementary material for: Powerful gene set analysis in GWAS with the Generalized Berk-Jones statistic
Source: PLoS Genet. 2019 Mar 15;15(3):e1007530. doi: 10.1371/journal.pgen.1007530 (PMC6436759; doi:10.1371/journal.pgen.1007530)
Supplement: S4 Table — Source refers to the pathway database holding the original entry, and ID is the identification number within that database. Note that there are still multiple ear development pathways at the top of the list, although Ear Morphogenesis is no longer significant after removal of FGFR2. The reason for this behavior is that the above ear development pathways include two highly significant genes and do not exhibit a large drop in p-value until we perform the step-down inference procedure with k = 2. Thus it can be useful to observe how results change as k is varied. (PDF) [file pgen.1007530.s012.pdf]

Description	Source	ID
Epithelial Cell Differentiation	GO Biological Process	GO:0002065
Inner Ear Development	GO Biological Process	GO:0048839
Ear Development	GO Biological Process	GO:0043583
MYD88 Toll-Like Receptor Signaling Pathway	GO Biological Process	GO:0002755
Fas Signaling Pathway	Panther	P00020
Positive Regulation Of Protein Autophosphorylation	GO Biological Process	GO:0031954
Regulation Of Protein Autophosphorylation	GO Biological Process	GO:0031952
TRAIL Signaling	NCI Pathway Interaction Database	TRAIL Signaling Pathway
Adenylate Cyclase-Activating GPCR Signaling	GO Biological Process	GO:0007189
TNFR1 Pathway	MSigDB C2	BioCarta TNFR1 Pathway
