# Supplementary material for: Powerful gene set analysis in GWAS with the Generalized Berk-Jones statistic
Source: PLoS Genet. 2019 Mar 15;15(3):e1007530. doi: 10.1371/journal.pgen.1007530 (PMC6436759; doi:10.1371/journal.pgen.1007530)
Supplement: S5 Table — Source refers to the pathway database holding the original entry, and ID is the identification number within that database. Note that the most significant pathways after removing the most significant gene are very different from the most significant pathways after removing the three most significant genes. In particular, ear development pathways are no longer significant. We recommend setting multiple different values of k in the step-down inference procedure to more fully understand the genetic etiology of different phenotypes. (PDF) [file pgen.1007530.s013.pdf]

Description	Source	ID
Oxidative Stress Induced Senescence	Reactome Database Id Release 59	2559580
G2 M Checkpoints	Reactome Database Id Release 59	69481
HDR Through Homologous Recombination Or SSA	Reactome Database Id Release 59	5693567
Fas Signaling Pathway	MSigDB	Fas Signaling Pathway
Lysophospholipid Pathway	MSigDB	Lysophospholipid Pathway
Homology Directed Repair	Reactome Database Id Release 59	5693538
Adenylate Cyclase-Activating GPCR Signaling	GO Biological Process	GO:0007189
Senescence-Associated Secretory Phenotype (SASP)	Reactome Database Id Release 59	2559582
LPA Receptor Mediated Events	NCI Pathway Interaction Database	LPA Receptor Mediated Events
Toll-Like Receptor Signaling Pathway	GO Biological Process	GO:0002224
