# Supplementary material for: Powerful gene set analysis in GWAS with the Generalized Berk-Jones statistic
Source: PLoS Genet. 2019 Mar 15;15(3):e1007530. doi: 10.1371/journal.pgen.1007530 (PMC6436759; doi:10.1371/journal.pgen.1007530)
Supplement: S6 Table — FGFR2 is the most significant gene in 169 of the pathways tested for association with breast cancer. After removing FGFR2, many of these pathways do not demonstrate the same strength of association. For some pathways, FGFR2 is the only gene in the entire set demonstrating strong association with breast cancer. After removing FGFR2, only 71 out of 169 pathways still demonstrate a p-value that is significant after correction for multiple testing. (PDF) [file pgen.1007530.s014.pdf]

	Original Pathways	Top Gene Removed	Top Three Genes Removed
Number Tested	169	169	163
Number $p < 1 \cdot 10^{-3}$	169	101	48
Number $p < 1 \cdot 10^{-5}$	169	73	14
Number $p < 4.65 \cdot 10^{-6}$	169	71	11
Number $p < 1 \cdot 10^{-10}$	169	49	0
