# Supplementary material for: Powerful gene set analysis in GWAS with the Generalized Berk-Jones statistic
Source: PLoS Genet. 2019 Mar 15;15(3):e1007530. doi: 10.1371/journal.pgen.1007530 (PMC6436759; doi:10.1371/journal.pgen.1007530)
Supplement: S7 Table — FGFR2 is the most significant gene in 169 of the pathways tested for association with breast cancer. These pathways are all highly associated with breast cancer. Many are still significant for association with height and schizophrenia, but it is no longer the case that all of them automatically exhibit p < 1 ⋅ 10−10. On one hand, it is desirable that a GSA test assigns high ranks to pathways with very significant genes. However, these genes can also dominate the analysis and produce results that are less useful to the researcher. (PDF) [file pgen.1007530.s015.pdf]

	Breast Cancer	Height	Schizophrenia
Number Tested	169	169	169
Number $p < 1 \cdot 10^{-3}$	169	152	128
Number $p < 1 \cdot 10^{-5}$	169	147	114
Number $p < 4.65 \cdot 10^{-6}$	169	145	102
Number $p < 1 \cdot 10^{-10}$	169	128	49
