# Supplementary material for: Powerful gene set analysis in GWAS with the Generalized Berk-Jones statistic
Source: PLoS Genet. 2019 Mar 15;15(3):e1007530. doi: 10.1371/journal.pgen.1007530 (PMC6436759; doi:10.1371/journal.pgen.1007530)
Supplement: S8 Table — In the step-down inference procedure, genes are removed based on their ranking in this list. For example, FGFR2 is often removed first because it is the 5th most significant gene overall. FGFR2 will only not be removed first if the pathway also contains NEK10, SLC4A7, CCDC91, or MAP3K1. Pathways with multiple genes at the top of this list generally show the most association with breast cancer, as the top genes can overwhelm data from the rest of the set. Such behavior motivates us to introduce the step-down inference procedure. (PDF) [file pgen.1007530.s016.pdf]

Rank	Gene	Number SNPs Used
1	NEK10	113
2	SLC4A7	90
3	CCDC91	150
4	MAP3K1	51
5	FGFR2	57
6	HNF4G	149
7	CASC8	195
8	KANSL1	131
9	STXBP4	87
10	DIRC3	206
11	EBF1	186
12	TOX3	50
13	MKL1	73
14	LINC02210-CRHR1	166
15	ASTN2	640
16	MAPT	111
17	HCN1	129
18	CASC21	56
19	MAPT-AS1	91
20	CCDC170	111
